# Brazilian Medical Survey on Telemedicine since the onset of COVID-19

**DOI:** 10.31744/einstein_journal/2023AE0428

**Published:** 2023-08-30

**Authors:** Eduardo Cordioli, Mara Giavina-Bianchi, Carlos Henrique Sartorato Pedrotti, Sérgio Podgaec

**Affiliations:** 1 Hospital Israelita Albert Einstein São Paulo SP Brazil Hospital Israelita Albert Einstein, São Paulo, SP, Brazil.

**Keywords:** Telemedicine, Health behavior, Telemonitoring

## Abstract

**Objective:**

The primary aim of this study was to understand the difference in the use of Telemedicine by Brazilian physicians before and after the onset of COVID-19 pandemic and their intention to continue using it post the pandemic period. The secondary objective was to analyze the differences of opinion between physicians in the private and public sectors.

**Methods:**

We conducted an online medical survey through the SurveyMonkey platform in a large hospital in São Paulo, Brazil, from May to July 2022.

**Results:**

Three-hundred-and -two physicians responded to the survey. We found that there was a significant increase in the number of physicians who started using Telemedicine in both the public and private sectors (p<0.0001) since the onset of COVID-19 pandemic and that >50% of them intend to continue using Telemedicine in their daily practice. Most responders consider that Telemedicine is useful in screening, diagnosis and management of patients; that it facilitates the physician’s daily practice; that it can maintain or even add financial gains through reducing office expenses; and that is another medium for dispensing medical care. They also believe that Telemedicine should be regulated by the Brazilian Federal Council of Medicine. There were no significant differences between the responses from private and public sector physicians.

**Conclusion:**

Telemedicine has played a major role in healthcare since the onset of COVID-19 pandemic and most of the physicians approve its use and intend to continue using Telemedicine in their daily practice.

## In Brief

Cordioli et al. showed in a survey, that there was a significant increase in the number of physicians using Telemedicine since the onset of the COVID-19 pandemic. Most of physicians believe it is useful, facilitates their practice and is another medium for dispensing medical care. They also intend to continue to use it in their daily medical practice.

## Highlights

Telemedicine played a major role in healthcare during the COVID-19 pandemic.

A statistically significant number of physicians started using Telemedicine since the onset of COVID-19 pandemic.

Most physicians approve its use and intend to continue using Telemedicine in their daily practice.

## Graphical abstract

**Figure:**
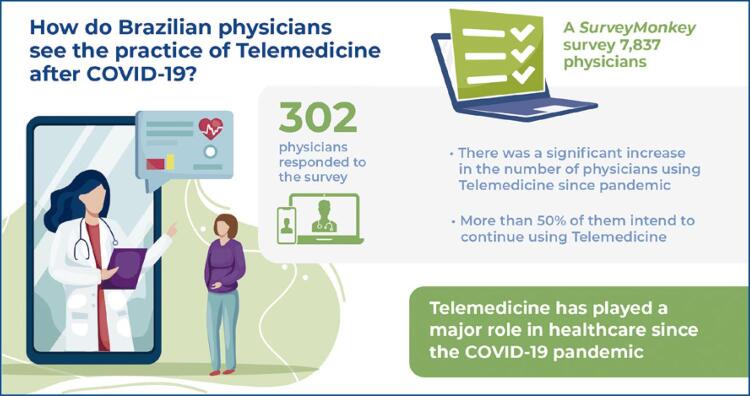

## INTRODUCTION

Telemedicine (TM) is defined by the World Health Organization as: “the promotion of health services by all healthcare professionals, where distance is a critical factor, using communication technologies to exchange valid information for diagnosis, treatment and prevention of diseases and injuries, as well as research and evaluations”.^(1)^ Telemedicine has increased patients’ access to healthcare,^(2)^ in addition to having shown high diagnostic accuracy.^(3,4)^However, several barriers, hinder the dissemination of its implementation,^(5,6)^ such as acceptance of the practice by the patient, by the physician, problems of connectivity, ethical, regulatory and privacy protection issues, in addition to reimbursement for the service.^(7-9)^

The COVID-19 pandemic began to spread in March 2020, and drastically changed the life of people around the world. The general recommendation adopted by most countries was to avoid leaving the house to prevent the spread of the disease. Thus, TM, previously adopted in more specific situations, such as for rural areas, long-distance consultations, or people with limited mobility, has become a form of care that was very necessary and sought by patients and physicians. Thus, the Brazilian Federal Council of Medicine (CFM - *Conselho Federal de Medicina*) had to propose an emergency change to expand the rules for the use of TM in that period, and, recently, a resolution with the number 2,314/2022 recognized the service TM rendered in the healthcare sector.^(10)^

Opinion surveys are important tools in assessing satisfaction in a particular service and consist of a list of questions whose objective is to extract certain data from a group of people.^(11)^ In our context, several international studies have shown high patient satisfaction, but there are not many studies seeking the opinion of physicians regarding the use of TM during the pandemic.^(8)^ There is scarce medical literature on the opinion of Brazilian physicians on this subject. Thus, we thought it was important to fill this gap.

## OBJECTIVE

To assess whether there was a significant difference in the use of Telemedicine by physicians before and after the beginning of the COVID-19 pandemic, and, if so, how often they intend to keep using Telemedicine in their daily routine. The secondary objective was to verify if there were differences in the opinions of physicians working in the public or private sectors regarding Telemedicine.

## METHODS

We undertook a cross-sectional observational study through an opinion survey approved by the Ethics Committee of *Hospital Israelita Albert Einstein* (HIAE), CAAE: 30749620.6.0000.0071; # 4.033.865. The target population were physicians from the clinical staff linked to HIAE. There were no exclusion criteria and no financial incentives to answer the questionnaire. The questionnaire contained 21 questions, and if the subject answered affirmatively to question number 21, an additional question (if all innovations such as laser, robotic or laparoscopic surgery should also be regulated by CFM) would be asked, totaling 22. Question number one was the informed consent form (ICF). Only after the ICF was provided, the next questions were presented to the individual. If not accepted, the survey would be terminated. The time required to complete the questionnaire was approximately 4 minutes, on average. The survey was completely anonymous and confidential, and only the authors of this study had access to the answers. The complete questionnaire is presented in (Supplementary Material 1). Its short version provided in tables 1 and 2. Table 1 shows questions and the answer options 2–13 and table 2, questions 14–22. It was sent by e-mail to all physicians with e-mail linked to HIAE, for those working in both the private and public sectors administered by HIAE. In the first email, a brief introduction inviting the physician to participate in the survey and the link of the questionnaire to be completed in the SurveyMonkey computer program (SurveyMonkey Inc., San Mateo, CA, USA; www.surveymonkey.com) were sent to 7,837 physicians. In the second round, we resent the same email to those, who according to the hospital’s Marketing Department, had not seen the previous one; *i.e*., it is, it was sent to 4,032 doctors again on 06/27/22. The survey was completed on 07/31/2022. To make sure that the same subject did not to respond to the survey more than once, there was a blocking mechanism present in the SurveyMonkey program that identifies and notifies the user that the questionnaire had already been answered. The research was previously tested on three physicians of the HIAE TM medical team, who were part of the test target population. Our work followed the guide to reporting CROSS survey studies (Checklist for Reporting Survey Studies).^(11)^

**Table 1:** Profile of physicians who answered the survey on Telemedicine: answers 2–13

Physicians	Public sector n=91 (%)	Private sector n=211 (%)	Total n=302 (%)
2. Sex (n=302)			
Female	38 (12.6)	86 (28.5)	124 (41.1)
Male	53 (17.6)	124 (41.1)	177 (58.6)
Not informed	0 (0.0)	1 (0.3)	1 (0.3)
3. Age in years (n=302)			
26–35	7 (2.3)	13 (4.3)	20 (6.6)
36–45	16 (5.3)	62 (20.5)	78 (25.8)
46–55	19 (6.3)	47 (15.6)	66 (21.9)
≥56	49 (16.2)	87 (28.8)	136 (45.0)
Not informed	0 (0.0)	2 (0.7)	2 (0.7)
4. Highest academic title (n=302) p=0.0004			
Medicine	1 (0.3)	4 (1.3)	5 (1.7)
Residency/Specialization internship	26 (8.6)	99 (32.8)	125 (41.4)
Master’s degree	22 (7.3)	56 (18.5)	78 (25.8)
PhD degree	24 (7.9)	42 (13.9)	66 (21.9)
Post-doc	5 (1.7)	4 (1.3)	9 (3.0)
Associate Professor	12 (4.0)	4 (1.3)	16 (5.3)
Other	1 (0.3)	2 (0.7)	3 (1.0)
5. Number of years since graduation (n=302) p=0.0330			
<5	1 (0.3)	2 (0.7)	3 (1.0)
5–10	6 (2.0)	18 (6.0)	24 (7.9)
11–20	11 (3.6)	56 (18.5)	67 (22.2)
>20	73 (24.2)	135 (44.7)	208 (68.9)
6. Specialization (n=302)			
Surgery	14 (4.6)	39 (12.9)	53 (17.5)
Internal Medicine	20 (6.6)	34 (11.3)	54 (17.9)
Dermatology	2 (0.7)	11 (3.6)	13 (4.3)
Management	2 (0.7)	6 (2.0)	8 (2.6)
Gynecology/Obstetrics	9 (3.0)	28 (9.3)	37 (12.3)
Ophthalmology	0 (0.0)	6 (2.0)	6 (2.0)
Orthopedics	8 (2.6)	10 (3.3)	18 (6.0)
Otorhinolaryngology	6 (2.0)	15 (5.0)	21 (7.0)
Other	14 (4.7)	33 (10.9)	47 (15.6)
Pathology	0 (0.0)	1 (0.3)	1 (0.3)
Pediatrics	12 (4.0)	20 (6.6)	32 (10.6)
Search	2 (0.7)	0 (0.0)	2 (0.7)
Psychiatry	2 (0.7)	6 (2.0)	8 (2.6)
Radiology	0 (0.0)	2 (0.7)	2 (0.7)
7. Do you work (n=302)			
Mainly in the private sector	0 (0.0)	211 (69.9)	211 (69.9)
Mainly in the public sector	18 (6.0)	0 (0.0)	18 (6.0)
Equally in both sectors	73 (24.2)	0 (0.0)	73 (24.2)
8 and 9. Workplace* (n=302)			
State of São Paulo	90 (29.8)	209 (69.2)	299 (99.0)
Other state or DF	2 (0.7)	3 (1.0)	5 (1.7)
Capital	91 (30.1)	206 (68.2)	297 (98.3)
Coast or inland	0 (0.0)	5 (1.7)	5 (1.7)
10. What is your opinion on TM? (n=288)			
A remote service, such as WhatsApp, SMS, email	58 (20.1)	123 (42.7)	181 (62.8)
Only a remote service with video communication	31 (10.8)	73 (25.3)	104 (36.1)
I do not know	0 (0.0)	3 (1.0)	3 (1.0)
11. Have you already been using TM before the onset of COVID-19? (n=289)			
Yes, through a specific platform	13 (4.5)	16 (5.5)	29 (10.0)
Yes, through WhatsApp, SMS, e-mail	44 (15.3)	120 (41.5)	164 (56.7)
No	33 (11.4)	62 (21.5)	95 (32.9)
I do not know	0 (0.0)	1 (0.3)	1 (0.3)
12. Have you been using TM since the onset of COVID-19? (n=288)			
Yes, through a specific platform	33 (11.5)	96 (33.3)	129 (44.8)
Yes, through WhatsApp, SMS, e-mail	44 (15.3)	86 (29.9)	130 (45.1)
No	12 (4.2)	17(5.9)	29 (10.1)
13. Intention to adopt TM if available and necessary? (n=289)			
Never	1 (0.3)	3 (1.0)	4 (1.4)
Rarely	10 (3.5)	21 (7.3)	31 (10.7)
Sometimes	33 (11.4)	69 (23.9)	102 (35.3)
Frequently	33 (11.4)	61 (21.1)	94 (32.5)
Always	13 (4.5)	45 (15.6)	58 (20.1)

* The value may exceed 100% because some doctors work in more than one state.

TM: telemedicine.

**Table 2:** Effects of Telemedicine on the day-to-day medical work: answers 14–22

Questions	Public sector	Private sector	Total	p value
14. Influence of TM in the number of appointments; n=288 (100%)				0.1230
Increases	43 (14.9)	102 (35.4)	145 (50.3)	
Reduces	8 (2.8)	7 (2.4)	15 (5.2)	
Stays the same	35 (12.2)	82 (28.5)	117 (40.6)	
Uncertain	4 (1.4)	7 (2.4)	11 (3.8)	
15. Does TM facilitate the work? (n=288)				0.3625
Yes	56 (19.4)	132 (45.8)	188 (65.3)	
No, it makes it more difficult	12 (4.2)	17 (5.9)	29 (10.1)	
No, it stays the same	16 (5.6)	44 (15.3)	60 (20.8)	
I do not know	5 (1.7)	6 (2.1)	11 (3.8)	
16. I believe TM (n=289)				0.7169
Is for screening only	19 (6.6)	56 (19.4)	75 (26.0)	
Is for diagnostics only	1 (0.3)	1 (0.3)	2 (0.7)	
Is for management only	3 (1.0)	9 (3.1)	12 (4.2)	
Is for diagnosis and management	48 (16.6)	100 (34.6)	148 (51.2)	
Has no utility	12 (4.2)	20 (6.9)	32 (11.1)	
It hinders medical care	3 (1.0)	12 (4.2)	15 (5.2)	
I do not know	4 (1.4)	1 (0.3)	5 (1.7)	
17. What is the role of TM in medical care? (n=289)				0.9596
Replaces face-to-face consultation	1 (0.3)	2 (0.7)	3 (1.0)	
Is one of the service options	84 (29.1)	184 (63.7)	268 (92.7)	
No change in in the day-to-day practice	4 (1.4)	9 (3.1)	13 (4.5)	
I do not know	1 (0.3)	4 (1.4)	5 (1.7)	
18. What do you think will be the impact of TM on financial gain? (n=289)				0.9514
Increases	30 (10.4)	66 (22.8)	96 (33.2)	
Decreases	11 (3.8)	29 (10.0)	40 (13.8)	
Stays the same	47 (16.3)	99 (34.3)	146 (50.5)	
I do not know	2 (0.7)	5 (1.7)	7 (2.4)	
19. What do you think will be the impact of TM on office expenses? (n=287)				0.6816
Expenses increase	7 (2.4)	21 (7.3)	28 (9.8)	
Expenses stay the same	16 (5.6)	40 (13.9)	56 (19.5)	
Expenses reduce due to fewer in-person follow-ups	40 (13.9)	70 (24.4)	110 (38.3)	
Expenses educe due to fewer in-person follow-ups and fewer on-site absences	13 (4.5)	30 (10.5)	43 (15.0)	
I do not know	14 (4.9)	36 (12.5)	50 (17.4)	
20. What should be done when there is disagreement between in-person and tele-physicians? (n=240)				0.2971
In-person’s opinion should prevail	34 (14.2)	76 (31.7)	110 (45.8)	
Tele-physician’s opinion should prevail	1 (4.2)	0 (0.0)	1 (4.2)	
A third opinion should be sought	32 (13.3)	83 (34.6)	115 (47.9)	
I do not know	6 (2.5)	8 (3.3)	14 (5.8)	
21. Do you believe that TM should be regulated by CFM? (n=289)				0.9257
Yes	78 (27.0)	175 (60.6)	253 (88.2)	
No	8 (2.8)	15 (5.2)	23 (8.0)	
I do not know	4 (1.4)	9 (3.1)	13 (4.5)	
22. If you responded “yes” to the previous question, do you believe all innovations such as laser, robotic and laparoscopic surgery in Medicine should be regulated as well? (n=274)				0.8710
Yes	72 (26.3)	164 (59.9)	236 (86.1)	
No	8 (2.9)	12 (4.4)	20 (7.3)	
I do not know	5 (1.8)	13 (4.7)	18 (6.6)	

Statistical analyses were performed using the χ^2^ test in Prism software version 6 (GraphPad Software, Inc., San Diego, CA, USA). The subjects were divided into a private or public sector based on the answer to question 7. Those who scored “mainly in the private sector” were included in the private group and those who scored “mainly in the public sector” or “equally in both sectors” were included in the public group. Completion rate was calculated by the number of surveys completed and sent/number of surveys initiated by respondents x 100. P value <0.05 was considered significant.

## RESULTS

The link for the questionnaire was sent to 7,837 physician’s email ids. The ICF was accepted by 312 physicians. The completion rate of the questionnaire was 93% (289/312). Table 1 (questions 2–13) shows physicians’ profile, their use of TM before and after the onset of COVID-19, and their willingness to adopt it in the future. The questionnaire would take approximately 4 minutes to complete. Majority of the respondents were men, aged above 56 years, working in the private sector, and have 20 or more years of experience since the completion of their graduation. Two-hundred-and -eleven respondents said that they work mainly in the private sector, 73 of them equally in both sectors, and 18 of them mainly in the public sector. We found that in the public sector, there are significantly more physicians with higher academic degrees and more number of years in training.

**Figure 1:**
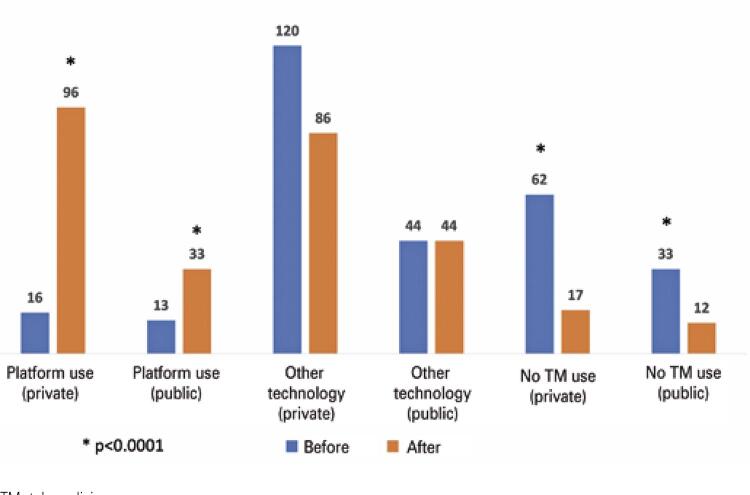
Use of Telemedicine before and after the beginning of the pandemic in the public and private sectors through a specific platform, using other technologies or its non-use

Two-hundred-and -eighty nine subjects were consistent in answering the questionnaire till the end. We found that 62.8% of the physicians consider TM a form of remote health service, while 36.1% have the opinion that TM is only online video communication. In both sectors, there was a significant increase in the use of TM since the beginning of the COVID-19 pandemic through platforms intended for that: from 29 (10.0%) to 129 individuals (44.6%; p<0.0001). In addition, there was a decrease in the number of physicians who did not use TM: from 95 (32.9%) to 29 (10.0%; p<0.0001). The number of individuals who used other technologies for TM remained stable in the public sector and decreased in the private sector (Figure 1). Data show that, currently, >50% of the physicians intend to continue using TM in their daily practice.

TM: telemedicine.

Regarding the daily routine shown in table 2 (questions 14–22), for the most part, physicians believe that TM increases the number of appointments, makes the work easier, is useful in the diagnosis and management of patients, is another medium for dispensing medical care, does not alter the financial remuneration, even reducing the office costs. There were no significant differences between the answers of physicians in the public and private sectors.

In the event of a disagreement between the physicians consulted in-person and through TM, there was a difference in the opinion on whose judgement should prevail. The responses were: “the opinion of the physician consulted in person must prevail” (110/239), and “a third opinion must be requested” (114/239). In this case, there was no difference between the opinions of the doctors belonging to either of the sectors (p=0.2971). Many physicians preferred to answer this question as a comment in an open field, such as: “mistakes can be made in both forms of service, but face-to-face consultation is more reliable”; “seek consensus”; “understand the reason for the disagreement”; “make medical decisions together with the patient”; “when in doubt, seek face-to-face consultation”.

The vast majority answered that TM should be regulated by the Brazilian Federal Council of Medicine (253/289). Of these, 236 out of 274 believe that other medical procedures, such as the use of laser, robotic and laparoscopic surgeries should also be regulated by the CFM.

## DISCUSSION

We conducted an opinion survey study on 302 physicians from a large hospital in the city of São Paulo, with a high completion rate. The results demonstrate the popularity that TM has acquired in recent years in the daily practice of physicians. It was already an interesting tool to dispense medical care in the areas with little access to physicians, either because of the physical distance, or because they were difficult to access, or due to limited availability of professionals. However, with the COVID-19 pandemic and the need of social distancing, TM has become an extremely useful and necessary tool. This was clearly confirmed by the significant increase in physicians who started using specific platforms for TM and by the number of physicians who did not use TM in their daily routine before the pandemic, but started to do so. Another important result is their clear intention to adopt the use of TM in their routine, even after the improvement in the pandemic situation. Knowing the favorable opinion of the physicians on TM in Brazil is very important to persuade different levels in the Brazilian government to implement TM as a routine method for dispensing medical care. It also corroborates the resolution issued recently by the CFM and helps the Legislative Power when it comes to ratifying the laws on the use of TM in Brazil. Moreover, as seen in the survey, physicians want TM to be regulated by the CFM, and also other medical procedures. This situation reflects the “clandestine” nature of TM before pandemics, which drove patients and physicians to risk, as there were no adequate and safe regulations for the TM practice in Brazil. After regulation, the use of specific platforms that meet the requirements established by CFM helps to ensure a safer TM practice. Furthermore, is important to highlight that regulation can prevent, unsafe activities, as well as underemployment in the medical field, which can be detrimental to both patients and physicians. As the technology services evolve quickly, it is essential that regulatory agencies, including CFM, have the structure and ability to rapidly judge and embrace such advances.

Telemedicine underwent a trial by fire during the pandemic, and had to be hastily adopted by many who had no intention to do so, at least in the short term; however, it was very well received. The survey shows that most physicians view TM very favorably. Since we had no previous surveys on this topic in Brazil; hence, these results would prove to be very helpful in planning health policies.

Other studies too have analyzed the acceptance of TM by physicians. In the case of asynchronous TM for primary care professionals, 83% of them considered the quality of teleconsultations as excellent or good, but, on the other hand, almost 60% said they had technical and organizational problems. These factors negatively influenced physicians’ intention to use the platform in the future.^(12)^Another study carried out with dermatologists before the pandemic showed high levels of satisfaction, with a significant increase in physicians’ confidence in the approach. However, this was also hampered by technical issues.^(13)^ In a survey conducted for general surgeons, <25% of them had come into contact with TM before the COVID-19 pandemic, and 95% of them reported interest in continuing to use it.^(14)^Our study shows a 4.5-fold jump in the number of physicians using TM through platforms before the pandemic. In Israel, 87% of physicians recognized the benefits of TM during the pandemic and 68% were in favor of continuing the services.^(8)^A survey in the United Kingdom with 96 primary care physicians showed 70% of the physicians believe in the contribution of TM in patient care.^(15)^ Another article reveals that 86% of the physicians planned to continue using TM after the pandemic^(16)^ while in our study it was 54%.

We found no significant differences between the private and public groups in the main responses to the questionnaire. However, it should be noted that the physicians surveyed in our study work mainly in the public sector, and these public hospitals are managed by HIAE, which have the possibility of performing TM. This may not be the reality of the Brazilian Unified Health System (SUS – *Sistema* Único *de Saúde*), in general. Therefore, if the same questionnaire were applied to physicians in the public health sector in different regions of the country, the answers could be very different, showing even a greater necessity and propensity to adopt TM. In fact, many may not even respond to our survey, as they never had access to the TM in their work.

In case of a possible disagreement between the physicians in a teleconsultation, many answers highlighted the role of face-to-face examination being fundamental in cases of doubts by the teleconsultants themselves or by another physician, in addition to dialogue for understanding the reason for disagreement, and setting up a multidisciplinary team when necessary. Involving the patient in the decision was also highlighted and we fully agree with all these statements. Telemedicine, to date, has obvious limitations, especially when physical examination is essential, and the patient will be directed to a face-to-face consultation in case of doubt or need. However, it has many benefits as already reported in other studies, such as the power to speed up diagnoses and treatments, prioritize critically ill or surgical patients, reduce the waiting list for specialists, since in many cases, TM can address less complex cases.^(2)^

A limitation of the study is that it was carried out in a single institution, bringing possible bias, especially because this hospital was one of the pioneers in TM in Brazil and has a department dedicated to its development. Another limitation of the study is that the results obtained cannot be generalized for all of Brazil.

We intend to expand the application of the questionnaire to more physicians under Brazilian Unified Health System, beyond the management of HIAE, and also, verify the perspective of patients, availing the services of both the private and public sectors, in order to also be able to compare the perception of TM from different viewpoints.

## CONCLUSION

The advent of the COVID-19 pandemic and the need of social distancing highlighted how Telemedicine has become extremely useful and necessary. This study clearly observed the significant increase in physicians who started using specific platforms meant for Telemedicine, and by the number of physicians who had never used Telemedicine in their daily routine, but started to do so, most of them with the intention to continue using it regardless of COVID-19 pandemic. Moreover, the greatly favorable medical opinion invites private and, more importantly, public managers, to expand this mode of healthcare.

## Supplementary material 1

**Table:** Questionnaire used for the survey to seek the opinion physicians on Telemedicine

1) Informed Consent Form:
Hello,
You are being invited to participate in an opinion survey on new technologies, such as Telemedicine, emerging in the health sector.
This study is being conducted by researchers from *Hospital Israelita Albert Einstein* (CAAE: 30749620.6.0000.0071; # 4.033.865) and aims to find out if participants are aware of these technologies, their use and the expectations they have about them.
There will be no direct benefits for the participants to undertake the survey, but the answers will contribute to a better understanding of Telemedicine and the need to disseminate more information in this regard.
All the answers are collected anonymously, hence, the participant can express themselves freely.
There is no financial reward or reimbursement of proven costs for participating in the research.
By clicking on the word "Accept", you will be directed to the link with the questions. If you do not want to participate, click on "I do not accept".
Accept
I do not accept
2) What is your sex?
Female
Male
I do not wish to reveal
3) How old are you?
18–25 years
26–35 years
36–45 years
46–55 years
>56 years
4) What is your highest level of education?
Degree in Medicine without Residency or Specialization Internship
Master's or MBA
PhD degree
Post-doc
Associated Professor
Other
5) Which of these areas do you fit in best?
Pediatrics
Internal Medicine
Surgery
Orthopedics
Gynecology and Obstetrics
Psychiatry
Ophthalmology
Otorhinolaryngology
Dermatology
Radiology
Pathology
Management
Research
Other
6) How many years has it been since you graduated?
<5 years
5–10 years
11–20 years old
>20 years
7) Where do you work as a physician:
Mainly in the public sector
Mainly in the private sector
Equally in both sectors (public and private)
8) Do you work in the state capital, or on the coast or inland?
Capital
Coast or Inland
9) Which state or Federal District? Please use the acronym
10) I consider that Telemedicine to be:
A remote service, whether synchronous (simultaneous) or Asynchronous (at different times)
Only a remote service with video communication
I do not know
11) Were you already using Telemedicine for your patients before the COVID-19 pandemic?
Yes, through a platform intended for this purpose
Yes, if we consider calls/messages via Whatsapp, SMS, telephone, e-mails
No
I do not know
12) Have you been using Telemedicine for your patients since the beginning of COVID-19 pandemic?
Yes, through a platform intended for this purpose
Yes, if we consider calls/messages via Whatsapp, SMS, telephone, e-mails
No
I do not know
13) How likely are you to adopt Telemedicine in your medical care routine, if available and if necessary?
Never
Rarely
Sometimes
Often
Always
14) Regarding the number of appointments on a day-to-day basis, do you believe that Telemedicine will:
Increase the number of appointments
Decrease the number of appointments
Not change the number of appointments
I do not know
15) Regarding the facilitation of your day-to-day work, do you believe that Telemedicine will:
Make your work easier
Make your work more difficult
Not change your work
I do not know
16) Regarding the utility of Telemedicine in your day-to-day work, do you believe that:
It only helps in the triage of cases
It only helps in diagnosis
It only helps in the conduct
It helps in diagnosis and management
It neither helps or hinders
It hinders medical care
I do not know
17) Regarding the type of medical work, do you believe that Telemedicine will:
Replace face-to-face consultation completely
Be another work option
Not change the work
I do not know
18) What do you think will be the impact of Telemedicine on financial gain?
Increase the earnings
Decrease the earnings
Does not change the earnings
I do not know
19) Assuming that there is discordance between the diagnosis or suggested conduct between the physician consulted in-person and the physician consulted by Telemedicine, what do you think should be done?
The opinion of the physician consulted in-person should prevail
The opinion of the teleconsulting physician must prevail
A third opinion must be requested
I do not know
20) Do you think that Telemedicine can reduce office costs?
No, on the contrary, it increases due to the use of the Telemedicine platform license
No, because license costs must be balanced with savings in face-to-face service expenses
Yes, because it will be possible to prepare the agenda and make follow-ups through home office
Yes, because in addition to the reasons above, the number of absences in the office should also be reduced.
I do not know
21) Do you believe that Telemedicine should be regulated by the Federal Council of Medicine?
Yes
No
I do not know
22) If you answered the previous answer as “yes”, do you think that all innovations in Medicine, such as robotic surgery, use of laser s, laparoscopic surgery, among others, should also be regulated?
Yes
No
I do not know
